# Identification and validation of interferon-stimulated gene 15 as a biomarker for dermatomyositis by integrated bioinformatics analysis and machine learning

**DOI:** 10.3389/fimmu.2024.1429817

**Published:** 2024-11-04

**Authors:** Xingwang Wang, Hao Hu, Guangning Yan, Bo Zheng, Jinxia Luo, Jianyong Fan

**Affiliations:** ^1^ Department of Dermatology, General Hospital of Southern Theater Command, Guangzhou, China; ^2^ Department of Radiation Therapy, General Hospital of Southern Theater Command, Guangzhou, China; ^3^ Department of Pathology, General Hospital of Southern Theater Command, Guangzhou, China

**Keywords:** dermatomyositis, integrated bioinformatics, machine learning, ISG15, biomarker

## Abstract

**Background:**

Dermatomyositis (DM) is an autoimmune disease that primarily affects the skin and muscles. It can lead to increased mortality, particularly when patients develop associated malignancies or experience fatal complications such as pulmonary fibrosis. Identifying reliable biomarkers is essential for the early diagnosis and treatment of DM. This study aims to identify and validate pivotal diagnostic biomarker for DM through integrated bioinformatics analysis and clinical sample validation.

**Methods:**

Gene expression datasets GSE46239 and GSE142807 from the Gene Expression Omnibus (GEO) database were merged for analysis. Differentially expressed genes (DEGs) were identified and subjected to enrichment analysis. Advanced machine learning methods were utilized to further pinpoint hub genes. Weighted gene co‐expression network analysis (WGCNA) was also conducted to discover key gene modules. Subsequently, we derived intersection gene from these methods. The diagnostic performance of the candidate biomarker was evaluated using analysis with dataset GSE128314 and confirmed by immunohistochemistry (IHC) in skin lesion biopsy specimens. The CIBERSORT algorithm was used to analyze immune cell infiltration patterns in DM, then the association between the hub gene and immune cells was investigated. Gene set enrichment analysis (GSEA) was performed to understand the biomarker’s biological functions. Finally, the drug-gene interactions were predicted using the DrugRep server.

**Results:**

Interferon-stimulated gene 15 (ISG15) was identified by intersecting DEGs, advanced machine learning-selected genes and key module genes from WGCNA. ROC analysis showed ISG15 had a high Area under the curve (AUC) of 0.950. IHC findings confirmed uniformly positive expression of ISG15, particularly in perivascular regions and lymphocytes, contrasting with universally negative expression in controls. Further analysis revealed that ISG15 is involved in abnormalities in various immune cells and inflammation-related pathways. We also predicted three drugs targeting ISG15, supported by molecular docking studies.

**Conclusion:**

Our study identifies ISG15 as a highly specific diagnostic biomarker for DM, ISG15 may be closely related to the pathogenesis of DM, demonstrating promising potential for clinical application.

## Background

Dermatomyositis (DM) is an autoimmune connective tissue disease characterized by distinct skin lesions (1). DM predominantly affects adults between 45 and 65 years of age, with a higher incidence in females and a prevalence of 6-7 per 100,000 (2). Diagnosis of DM is challenging, especially when the classical skin symptoms (such as Gottron’s papules, Gottron’s sign, and heliotrope rash) or muscle weakness are absent (3). The early diagnosis is crucial, as approximately 30% of individuals with DM develop an associated malignancy (4). Meanwhile, DM usually associated with fatal complications such as pulmonary fibrosis or the myositis that may lead to permanent damage (2).

DM is known to have a significant genetic basis, yet the precise mechanisms underlying its pathogenesis remain unclear (5). The advent of high-throughput genomic technologies has facilitated a detailed examination of gene expression across various tissues, leading to the identification of numerous potential biomarkers (6, 7). Notably, a significant finding in DM patients is the marked upregulation of the type I interferon (IFN) pathway in tissues such as muscle, skin and blood (8, 9). This upregulation correlates with cutaneous disease activity in adult DM, evidenced by a type I IFN gene signature, suggesting their potential role in the disease’s progression (8, 10). Among these genes, ISG15 stands out as a ubiquitin-like modifier that is strongly induced by type I IFN (8, 9). Studies conducted by Preuße and Salajegheh have consistently shown a significant upregulation of ISG15 transcripts and proteins in DM muscle biopsy samples compared to samples from other inflammatory myopathies and healthy controls (11, 12). This upregulation is particularly pronounced in DM with perifascicular atrophy (PFA), suggesting that ISG15 may play a critical role in the development or progression of PFA, a characteristic and specific DM muscle lesion (12). Furthermore, ISG15 is implicated in critical biological processes, including antiviral defense, immune regulation, and possibly tumorigenesis (13). Despite advancements in the understanding of DM, early and accurate diagnosis remains a significant challenge. This is due to the variability in clinical presentation and similarities with other skin diseases, such as seborrheic dermatitis and rosacea (2, 14). These situations often lead to misdiagnoses and diagnostic delays. Traditional diagnostic criteria, such as those proposed by Bohan and Peter in 1975 (15, 16) or the European League Against Rheumatism/American College of Rheumatology (EULAR/ACR) criteria (17), depend on a combination of clinical signs, histological analysis of muscle biopsies, and laboratory tests associated with myositis, like creatine kinase (CK) and lactate dehydrogenase (LDH). However, these criteria sometimes lack sensitivity, particularly for forms of DM without muscle weakness, known as amyopathic DM (18). Moreover, muscle biopsies can be traumatic, frequently meet with patient reluctance, and are not always ideal for early detection. This highlights a need for biomarkers that are less invasive while sensitive and convenient, enabling the early detection of DM, even if only to provide effective indications. It is therefore critical to identify accurate molecular targets associated with the onset and progression of DM to improve diagnostic and treatment strategies.

In this study, we employed a systematic approach to identify potential biomarkers for DM. Utilizing a combination of bioinformatics and machine learning techniques, we analyzed a comprehensive dataset to pinpoint ISG15 as a key molecule of interest. Our findings suggest that ISG15 plays a critical role in the pathogenesis of DM, making it a promising target for diagnostic and therapeutic strategies.

## Materials and methods

### Data acquisition and data preprocessing

**Figure 1:**
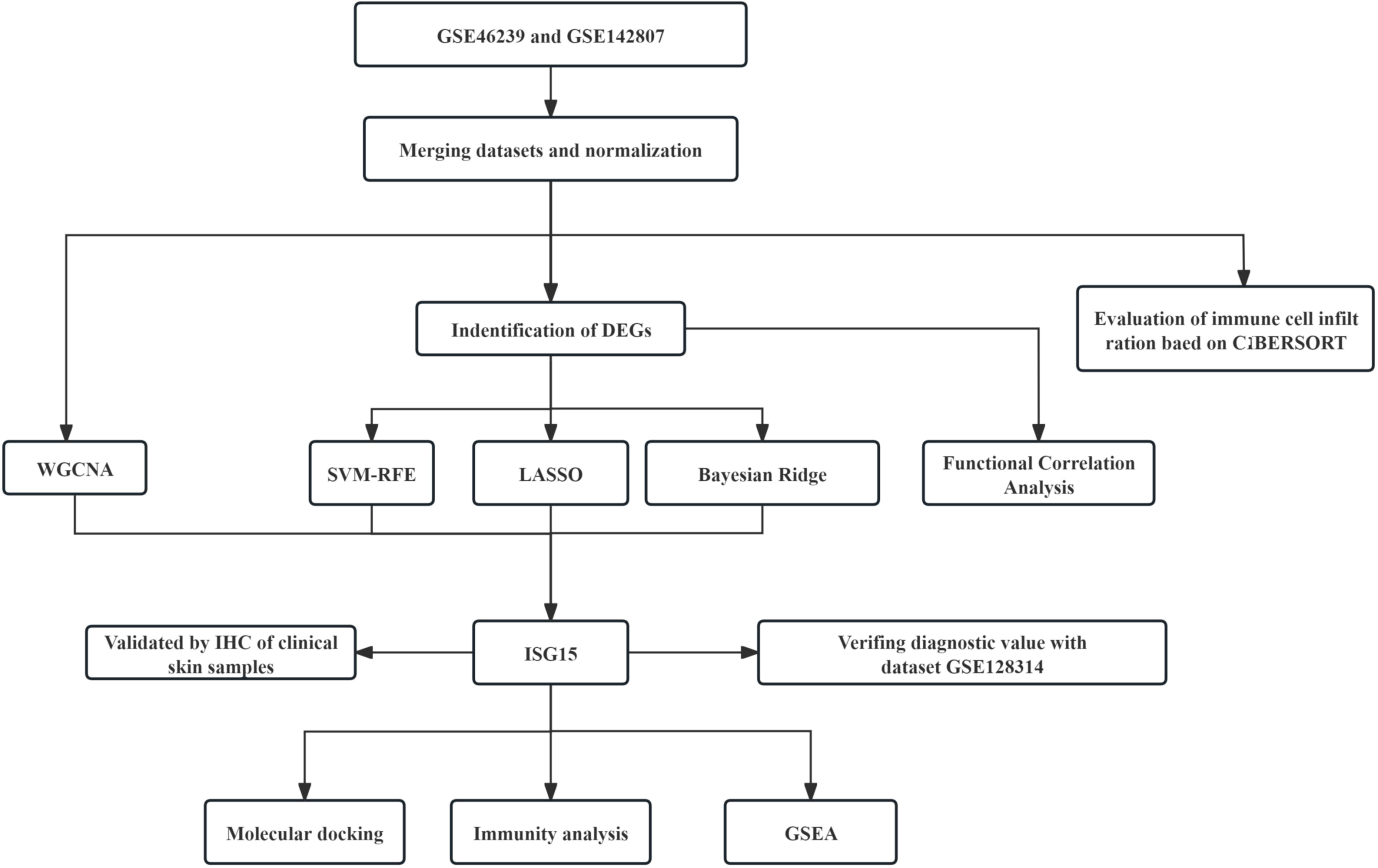
Schematic diagram illustrating the comprehensive bioinformatics analysis and validation methodology for DM research.

Our study’s workflow was depicted in **Figure 1**. Datasets in GEO (https://www.ncbi.nlm.nih.gov/geo/) database were accessed, the selection criteria included: 1) Adequate sample size to provide statistical power. 2) Availability of raw expression data. 3) Inclusion of both DM and control skin samples for comparative analysis. Based on these criteria, we accessed datasets (GSE46239, GSE142807 and GSE128314) containing gene expression data in skin lesion samples of DM patients and controls. The GSE46239 and GSE142807 datasets, which offer a larger sample size, were selected and merged for the analysis discovery phase to ensure that our machine learning models could sufficiently learn. In contrast, the GSE128314 dataset, having a smaller sample size, was utilized for the validation phase to test the model’s performance and generalization ability on an independent dataset.

We initially applied the “inSilicoMerging (19)” R package to merge the GSE46239 and GSE142807 datasets into a single combined dataset. To correct for batch effects in the merged dataset, we used the “removeBatchEffect” function from the “limma” R package (version 3.42.2). The batch effect-corrected expression matrix was then used for the identification of DEGs.

### Differential expression analysis and functional correlation analysis

The selection of DEGs was performed using the “limma” package in R, adhering to the criteria of an |LogFC| > 1 and adj.p < 0.05. Then these DEGs underwent Gene Ontology (GO) and Kyoto Encyclopedia of Genes and Genomes (KEGG) pathway analyses, utilizing the “org.Hs.eg.db” R package and the KEGG REST API for annotations. The maximum gene set is 5000 and the minimum gene set is 5.

### Machine learning for feature selection

After identifying DEGs, we applied three machine learning algorithms to further refine potential biomarker selection. The algorithms included the Least Absolute Shrinkage and Selection Operator (LASSO) (20), Support Vector Machine-Recursive Feature Elimination (SVM-RFE) (21), and Bayesian Ridge regression (22). The “glmnet” and “caret” R packages were used for LASSO and SVM-RFE, respectively. Sklearn in Python was used to conduct Bayesian ridge analysis. These methods are known for their efficacy in handling high-dimensional data and aiding in biomarker discovery.

### Weighted gene co-expression network analysis

To investigate gene correlations, WGCNA (23) package in R was applied. Initially, we calculated the median absolute deviation (MAD) for each gene, subsequently excluding the lower 50% based on MAD values. Next, “goodSamplesGenes” function was used to remove unsuitable genes and samples, followed by the construction of a scale-free co-expression network. The network’s adjacency was established using a “soft” thresholding power (β) based on co-expression similarity. Specifically, Pearson’s correlation matrices and the average linkage method were performed for all pairwise genes. A weighted adjacency matrix was then constructed using a power function A_mn=|C_mn|^β (C_mn = Pearson’s correlation between Gene_m and Gene_n; A_mn= adjacency between Gene m and Gene n. The soft-thresholding parameter β was selected to emphasize strong correlations between genes and penalize weak ones. After choosing the power of 8, the adjacency was transformed into a topological overlap matrix (TOM), which measures a gene’s network connectivity as the sum of its adjacency with all other genes. Subsequently, the corresponding dissimilarity (1-TOM) was calculated. For the classification of genes into modules based on similar expression profiles, average linkage hierarchical clustering was conducted using the TOM-derived dissimilarity measure, with a minimum cluster size of 30 genes for the gene dendrogram. Further analysis included the calculation of dissimilarity among module eigengenes, selection of a cut height for the module dendrogram, and merging of selected modules. Additionally, modules with a distance less than 0.25 were merged, ultimately resulting in 10 co-expressed modules.

### Validation using independent dataset

We intersected DEGs, machine-learning-selected genes, and WGCNA key module genes to identify potential biomarkers. The finding was presented in a Venn diagram, which was drawn using the Venn online tool (http://soft.sangerbox.com/). For validation purposes, the independent dataset GSE128314 was utilized. This dataset includes RNA expression data extracted from the skin tissues of eight DM patients and five healthy controls. To verify the predicted value of the biomarkers, we constructed a logistic regression model using “PROC” package in the R. The diagnostic value of the identified biomarkers was assessed by the area under the ROC curve (AUC, AUC was between 0.5 and 1). The closer the AUC is to 1, the more effective the diagnosis is. Additionally, a controlled reliability analysis was performed to statistically compare gene expression differences between DM patients and healthy controls using the same dataset.

### Human samples

All participants diagnosed with DM and healthy controls provided informed consent in the study. The study’s experimental protocols were rigorously reviewed and received approval from the Ethics Committee of the General Hospital of Southern Theater Command of the PLA, ensuring compliance with ethical standards.

### Immunohistochemistry

IHC was performed on skin biopsy specimens from 9 DM patients and 12 healthy controls to validate our bioinformatics findings. The paraffin-embedded tissue sections were first deparaffinized in xylene and subsequently rehydrated through a graded ethanol series. Antigen retrieval was carried out using 1 mM EDTA, followed by a preincubation with 5% goat serum in Tris-buffered saline to minimize non-specific interactions. The sections were then probed with a rabbit monoclonal anti-ISG15 antibody (EPR24482-49, at a 1:500 dilution). Following the application of horseradish peroxidase-conjugated secondary antibodies, visualization was achieved with 3,3’-diaminobenzidine substrate, and the sections were counterstained with hematoxylin for contrast.

### Evaluation of immune cell infiltration, correlation analysis between biomarker and infiltrating immune cells

To assess the extent of immune cell infiltration and its relationship with biomarker in DM, we employ the CIBERSORT algorithm (24) to interpret the relative abundance of 22 distinct immune cell types within the tissue. Furthermore, we generate a correlation heatmap to illustrate the interrelationships among different immune cell subsets within each sample. Comparative analysis of immune cell profiles between DM and healthy tissues is also conducted and presented visually. Next, we examined and graphically depicted the correlations between the levels of infiltrating immune cells and the proposed diagnostic biomarker using Spearman’s correlation coefficient.

### Gene set enrichment analysis

GSEA was employed to identify the involvement of ISG15 in biological processes and KEGG pathways related to DM. We stratified the samples into two groups based on ISG15 expression: low expression group (<50%) and high expression group (≥50%). Reference gene sets from the Molecular Signatures Database were utilized, namely c5.go.bp.v7.4.symbols.gmt for GO terms and c2.cp.kegg.v7.4.symbols.gmt for KEGG pathways. To assess statistical significance, we compared the enrichment scores to those from 1000 permutations of the dataset. NOM p <0.05 and |NES|>1 was considered significant enrichment.

### Virtual screening for drug repurposing

The molecular structure of target protein was sourced from the Protein Data Bank (PDB) database (https://www.rcsb.org/). Virtual screening was performed using the DrugRep server (http://cao.labshare.cn/drugrep/), an online tool that integrates the AutoDock Vina algorithm to dock multiple ligands. DrugRep evaluates and ranks the best-fitting molecular conformers by calculating their docking scores, binding conformations, and affinities with the receptor. The server’s receptor-based screening approach identifies potential binding sites based on the receptor’s 3D structure and conducts high-throughput docking using libraries of FDA-approved drugs, experimental drugs, and traditional Chinese medicine (TCM) compounds.

### Statistical analysis

Statistical analysis was conducted using the R and Python software. The significance of differential gene expression was ascertained using adjusted p-value to correct for the multiple testing phenomenon, with a significance threshold set at p-value < 0.05.

## Result

### Data preprocessing and identification of DEGs

**Figure 2:**
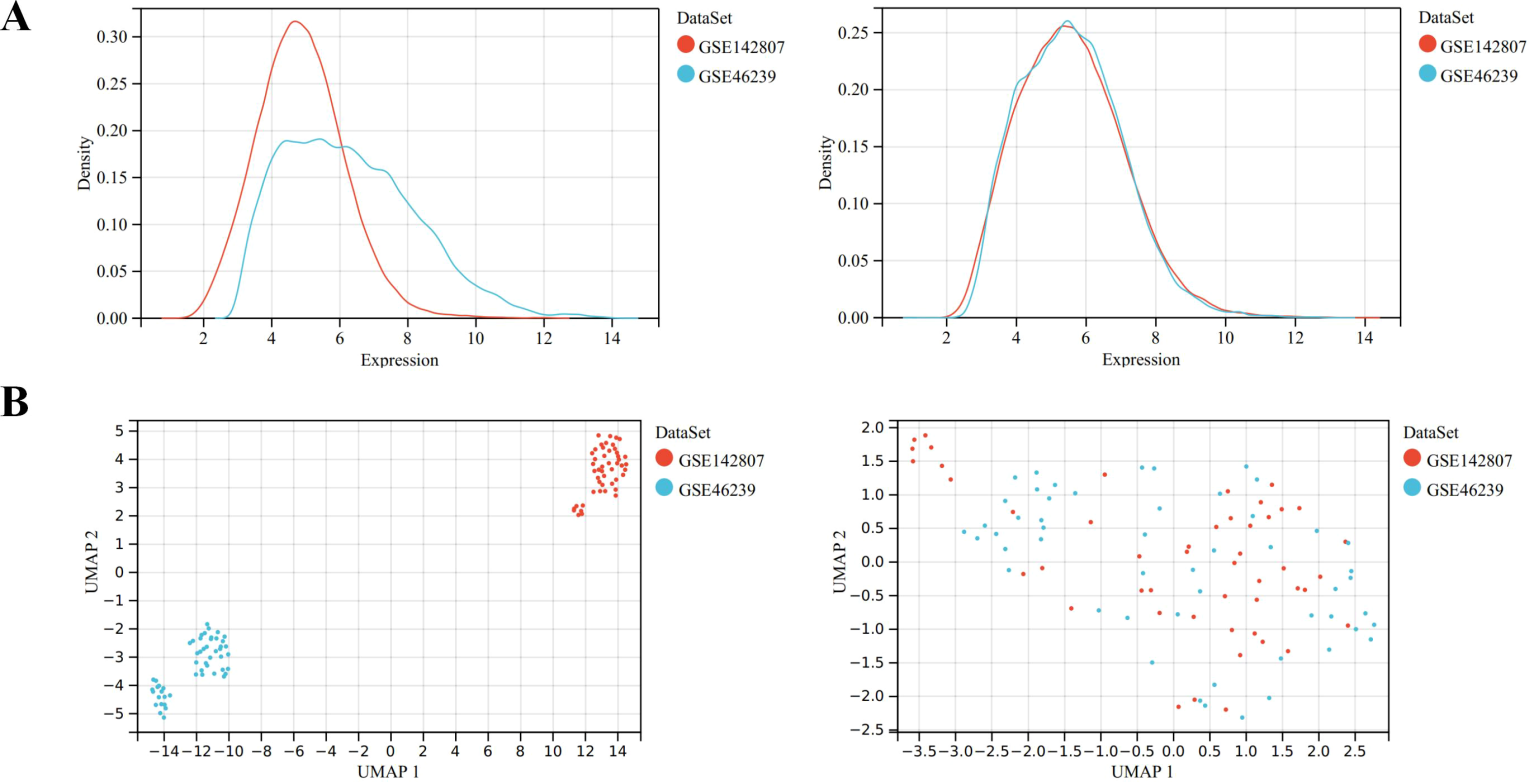
Data preprocessing of GSE46239 and GSE142807. **(A)** Density map showing the sample distribution of each data set before batch correction and after batch correction. **(B)** UMAP analysis showing the sample distribution of each data set before batch correction and after batch correction.

**Figure 3:**
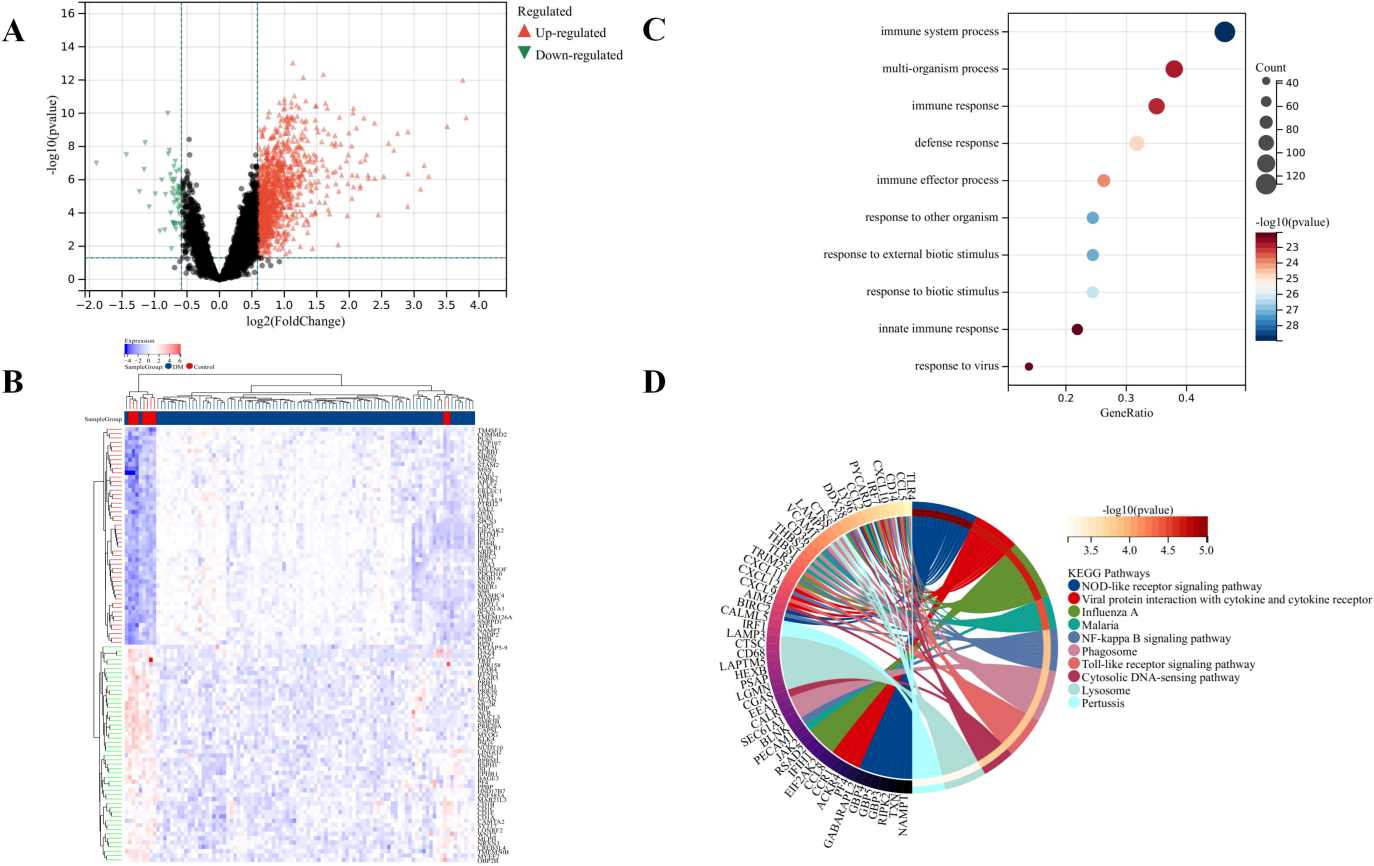
Identification of DEGs for DM. **(A)** Volcano plots showing DEGs between DM and normal group, red and green plot triangles represent DEGs with upregulated and downregulated gene expression, respectively. **(B)** Cluster heatmap showing the top 50 significantly upregulated and down-regulated DEGs, each row shows the DEGs, and each column refers to one of the samples of DM cases or controls. The red and blue represent DEGs with upregulated and downregulated gene expression, respectively. **(C)** Top 10 of GO biological processes analysis. **(D)** Top 10 of KEGG pathway analysis.

Microarray data GSE46239 and GSE142807 were obtained from the GEO database, including samples from 91 DM patients and 9 normal controls. After merging the two datasets, batch-to-batch variance was removed from the matrix of gene expression. The density plots in **Figure 2A** show substantial differences in sample distributions across datasets prior to batch effect removal, indicating the presence of batch effects. After correcting for batch effects, the data distributions among the datasets became more consistent, with similar means and variances. **Figure 2B** depicts UMAP results for the multiple datasets, with different colors representing different datasets before batch effect removal. Initially, the two datasets do not overlap, indicating their independence. Following the removal of batch-to-batch variance, the sample distributions between datasets became more uniform. After preprocessing the data, we extracted the DEGs in the gene expression matrix. Under the criteria of an |LogFC| > 1 and adj.p < 0.05, 292 genes with significant differential expression were identified in DM cases: 286 were up-regulated and 6 down-regulated. **Figures 3A, B** display a volcano plot of DEGs and a heatmap of the top 50 DEGs, respectively.

### Function enrichment analysis of the DEGs

The GO and KEGG analyses results demonstrated the involvement of the DEGs in immune response and inflammation. Specifically, the GO analysis highlighted broad biological processes such as immune system processes and defense responses (**Figure 3C**). In contrast, the KEGG analysis provided a detailed view of specific signaling pathways, including NOD-like receptor signaling and NF-kappa B signaling, which are critical components of the innate immune and inflammatory responses (**Figure 3D**). These findings suggest a coordinated regulation of immune and inflammatory responses at both the process and pathway levels by the DEGs in DM cases. In particular, The GO terms “immune system processes” and “defense responses” indicate that the DEGs are likely involved in a broad array of immune activities, encompassing both innate and adaptive immunity, consistent with the immunopathological nature of DM. These terms also suggest that the DEGs may influence defense mechanisms against infections, inflammation, and tissue damage.

In KEGG analysis, the enrichment of critical immune and inflammatory pathways such as the “NOD-like receptor signaling” and “NF-kappa B signaling” pathways was noted. The NOD-like receptor signaling pathway plays a key role in pathogen recognition and inflammation regulation, while NF-kappa B signaling is pivotal in controlling immune responses and inflammation. The enrichment of these pathways suggests that our DEGs might influence the disease mechanism of DM by modulating the activity of these pathways. Overall, the GO and KEGG analysis results provide compelling evidence that the DEGs may have a significant impact on the pathogenesis of DM by regulating immune and inflammatory responses.

### Feature selection through machine learning

**Figure 4:**
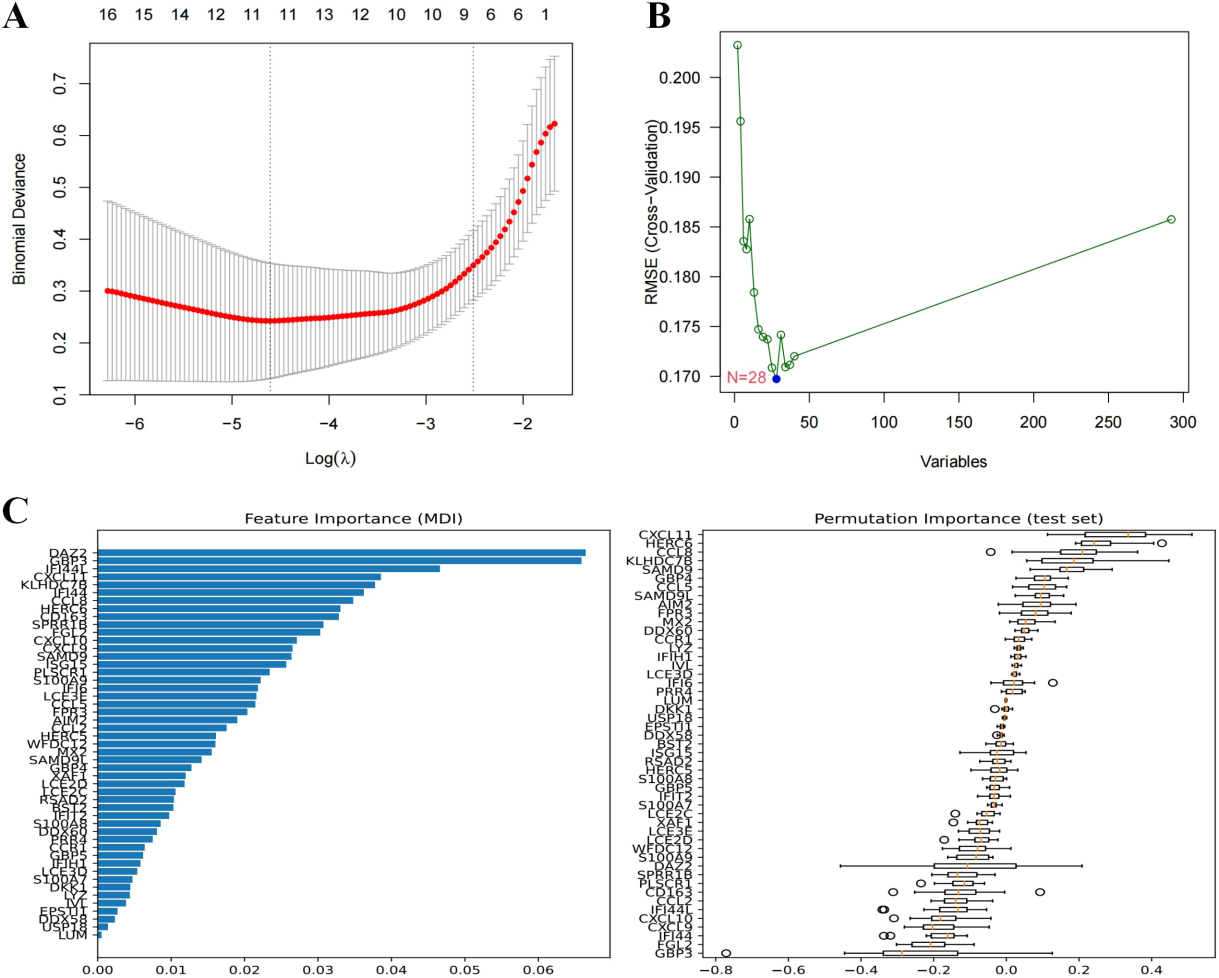
Machine learning in screening candidate diagnostic biomarkers for DM. **(A)** Biomarkers screening in the Lasso model. The number of genes (n=11) corresponding to the lowest point of the curve is the most suitable for DM diagnosis. **(B)** Based on SVM-RFE to screen biomarkers. **(C)** Biomarkers identified by Bayesian Ridge, genes are ranked based on the importance score.

Machine learning algorithms includes LASSO regression, SVM-RFE, and Bayesian ridge were employed to further refine the selection of candidate genes. LASSO regression identified 11 genes, SVM-RFE ranked 28 genes by importance, and Bayesian ridge highlighted 49 significant genes, as shown in **Figures 4A–C**.

### Weighted gene co-expression network construction and identification of clinically significant modules

**Figure 5:**
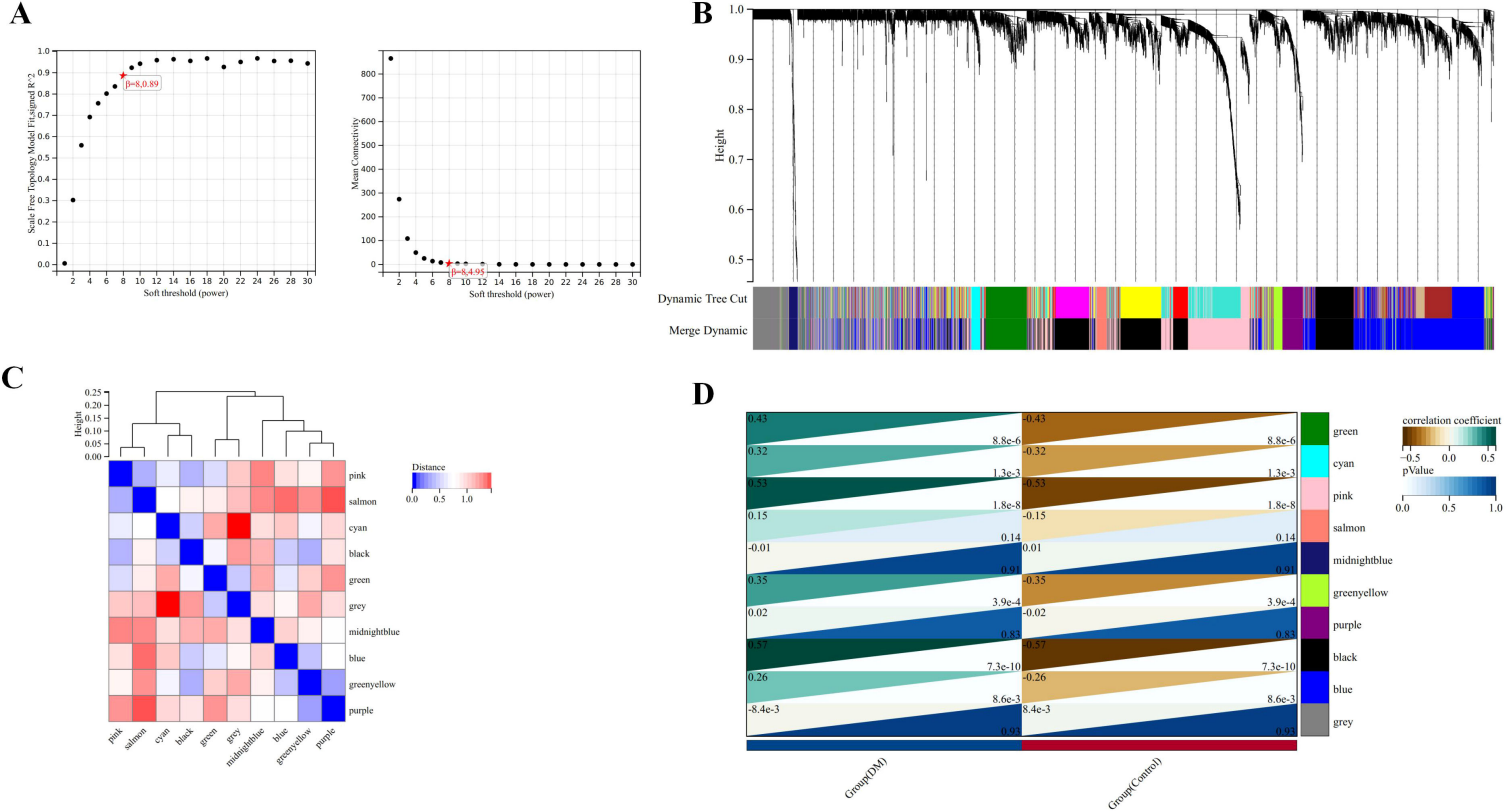
Identification of modules associated with the clinical traits of DM based on WGCNA analysis. **(A)** The soft threshold power (left) and mean connectivity (right) of WGCNA network. **(B)** Gene co-expression modules represented by different colors under the gene tree. **(C)** Clustering heatmap of module feature vector. **(D)** Heatmap of the correlation between module eigengenes and clinical traits of DM.

A “soft” threshold of β=8 (scale-free, R^2^ = 0.89) was chosen based on scale independence and mean connectivity (**Figure 5A**). Ten gene co-expression modules (GCMs) were identified, represented by different colors in **Figures 5B, C**. The correlation between DM and GCMs is shown in **Figure 5D**. Modules with a correlation coefficient > 0.5 and a p < 0.05 were selected for further analysis. Specifically, the black module (correlation coefficient=0.57, p=7.3e-10) and the pink module (correlation coefficient=0.53, p=1.8e-8) were selected for further analysis. Within these modules, 178 key genes were identified based on high connectivity, using a cutoff criterion of module membership (MM) > 0.8 and gene significance (GS) > 0.1.

### Validation of ISG15 as a biomarker for DM

**Figure 6:**
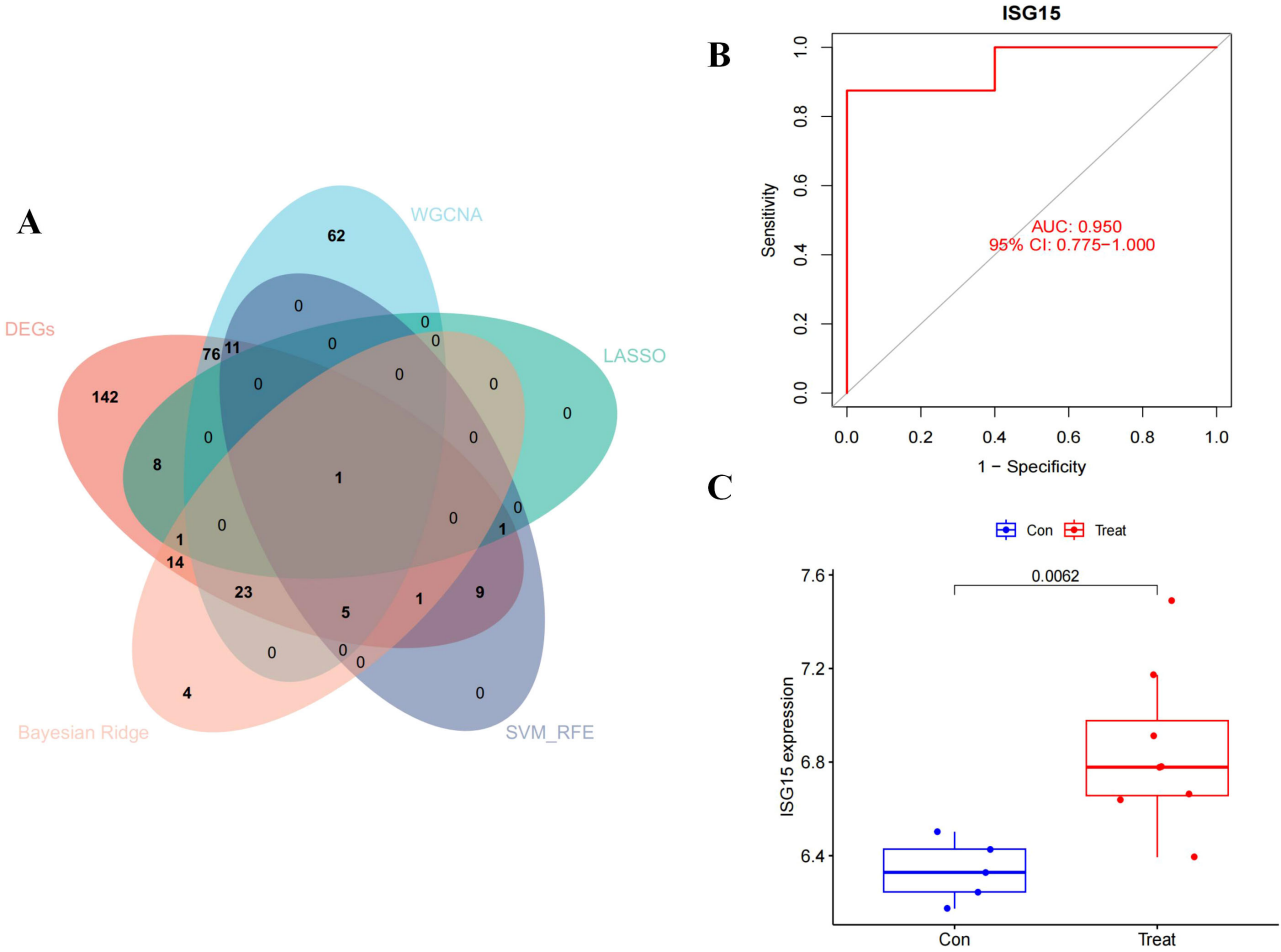
Diagnostic effectiveness and dataset validation of the hub gene for DM. The Venn diagram of the intersection of DEGs, module genes of WGCNA, LASSO, SVM-RFE and Beyasian ridge analysis. **(B)** ROC curve to assess the diagnostic efficacy of ISG15. **(C)** Data validation of ISG15 by GSE128314.

ISG15 was identified by intersecting the genes filtered through the aforementioned methods, as illustrated in the Venn diagram (**Figure 6A**). An independent validation dataset analysis confirmed the overexpression of ISG15 in DM patients. ROC curve analysis of ISG15 expression yielded an AUC of 0.950, with a 95% CI of 0.775-1.000 (**Figure 6B**), indicating excellent diagnostic performance. Furthermore, ISG15 expression was significantly higher in DM compared to the control group (**Figure 6C**).

### IHC of ISG15 expression

**Figure 7:**
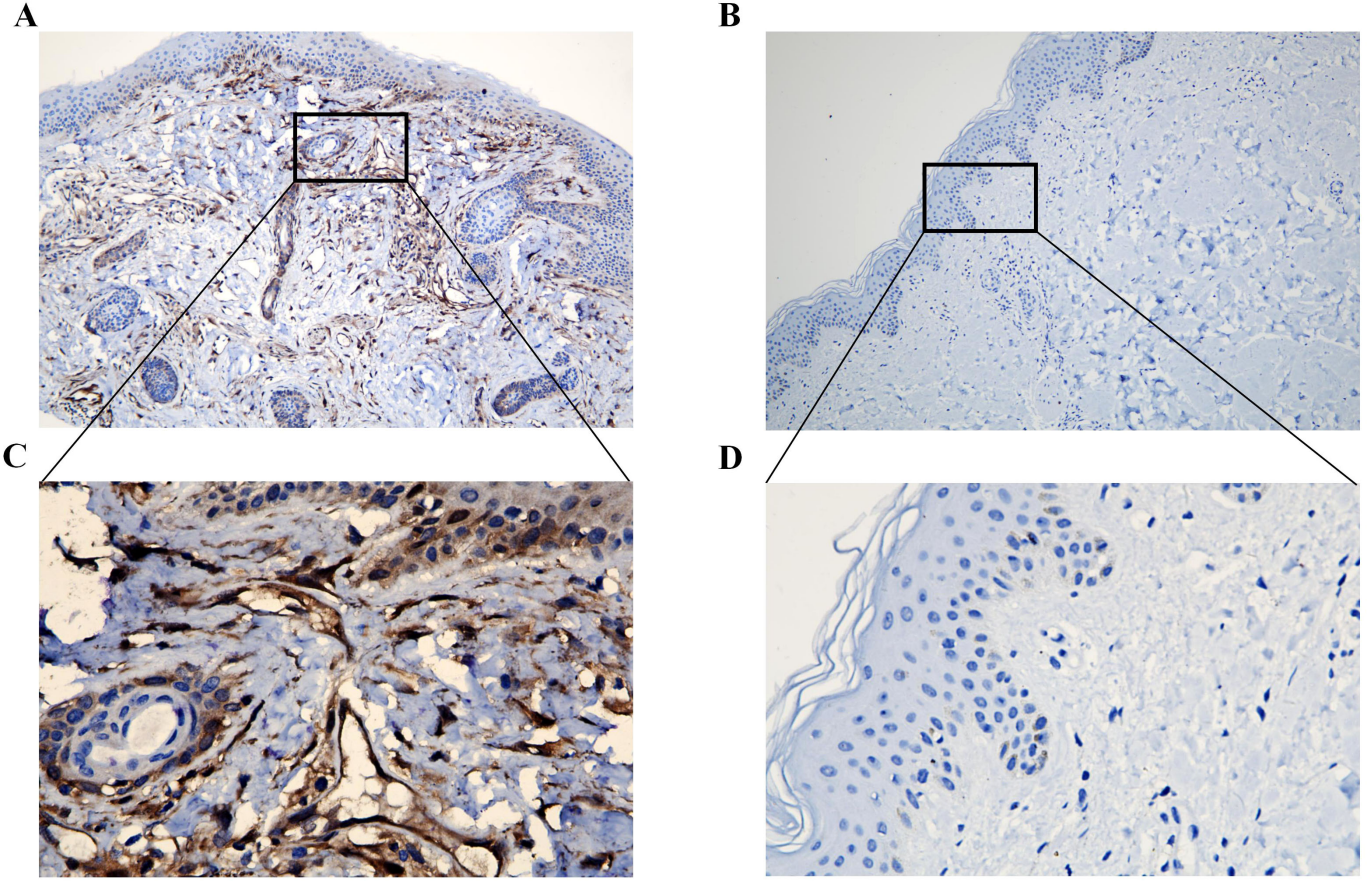
Immunohistochemical analysis of ISG15 expression in clinical skin samples. **(A)** Immunohistochemical analysis of ISG15 expression in DM group (X10). **(B)** Immunohistochemical analysis of ISG15 expression in control group (X10). **(C)** Immunohistochemical analysis of ISG15 expression in perivascular regions and lymphocytes of DM group (X40). **(D)** Immunohistochemical analysis of ISG15 expression in control group (X40).

IHC of clinical skin biopsy specimens from the DM group revealed universally positive expression of ISG15, in contrast to the negative expression observed in the control group (**Figures 7A, B**). Additionally, the immunostaining was primarily observed in the perivascular regions and lymphocytes within the skin tissue (**Figure 7C**).

### Immune cell infiltration analysis

**Figure 8:**
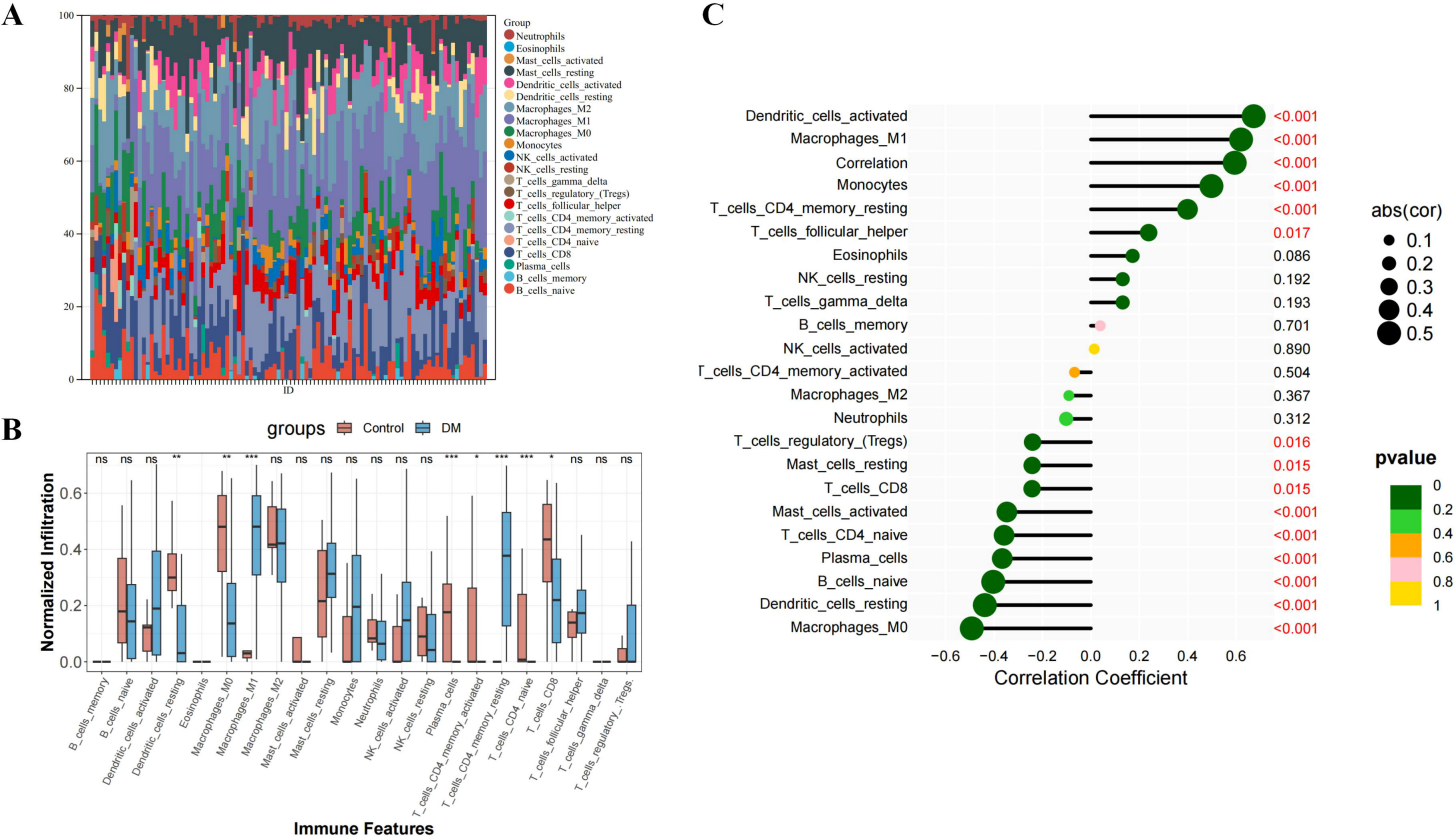
Immune infiltration analysis of DM. **(A)** The ratio of 22 immune cells of each sample of DM. **(B)** The immune cell infiltration between DM and normal controls. **(C)** The association between ISG15 and different immune cell infiltration in DM. *: P<0.05, **: P<0.01, ***: P<0.001 and term “ns” means no significance (P>0.05).

We employed CIBERSORT to assess the infiltration of 22 immune cell types in DM compared to normal tissues. The histograms in **Figure 8A** display the proportional representation of these cells in each sample, with the sum of proportions in each histogram equaling 100%. Our analysis identified that M1 macrophages, M2 macrophages, and resting mast cells were present in all DM samples. T follicular helper cells and CD8 T cells were also highly prevalent, found in 88% and 87% of the samples, respectively. **Figure 8B** presents a comparative analysis that shows a notable increase in several immune cell types in DM tissues versus controls, including resting dendritic cells, M0 macrophages, plasma cells, activated memory CD4 T cells, CD8 T cells, and naive CD4 T cells. This indicates a unique immune profile in DM, characterized by the involvement of both innate and adaptive immune responses in the tissue pathology.

### Correlation between ISG15 and immune cells

Spearman correlation analysis was conducted to explore the relationship between ISG15 expression and immune cell abundance. **Figure 8C** reveals statistically significant negative correlations between ISG15 expression and several immune cell populations, including resting dendritic cells, M0 macrophages, activated and resting mast cells, plasma cells, naive CD4 T cells, CD8 T cells and naive B cells. In contrast, M1 macrophages, monocytes, resting memory CD4 T cells, T follicular helper cell and dendritic cells exhibited a positive correlation. These findings suggest that ISG15 may play diverse roles in regulating various immune cells within the DM microenvironment, potentially influencing the functions and abundance of these cells and further driving the pathologic processes of DM.

### GSEA of ISG15

**Figure 9:**
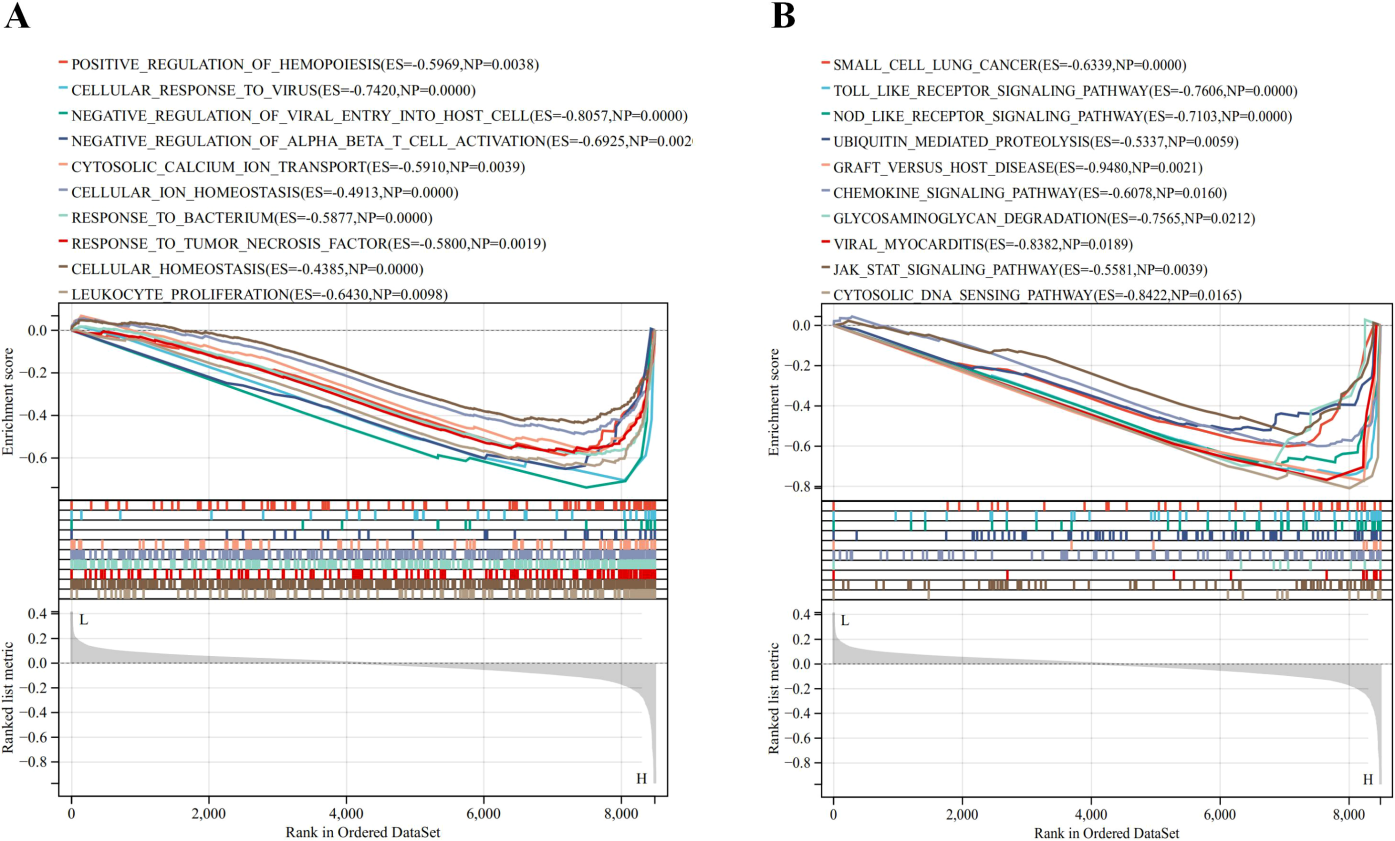
Result of Gene Set Enrichment Analysis of ISG15. **(A)** Biological processes enriched by ISG15. **(B)** KEGG pathways by ISG15.

The GSEA method was employed to explore the biological processes and signaling pathways affected by ISG15 expression in DM. The analyses reveal that ISG15 is implicated in key immune and inflammatory pathways, including the toll-like and NOD-like receptor signaling pathways, the JAK-STAT signaling pathway and ubiquitin-mediated proteolysis (**Figure 9A**). These pathways are essential for initiating and regulating immune responses, maintaining cellular homeostasis, and controlling the proliferation and activation of immune cells (**Figure 9B**). These analyses underscore the multifaceted role of ISG15 in the immune system and its potential impact on various biological processes.

### Drug-gene interaction and molecular docking analyses

**Figure 10:**
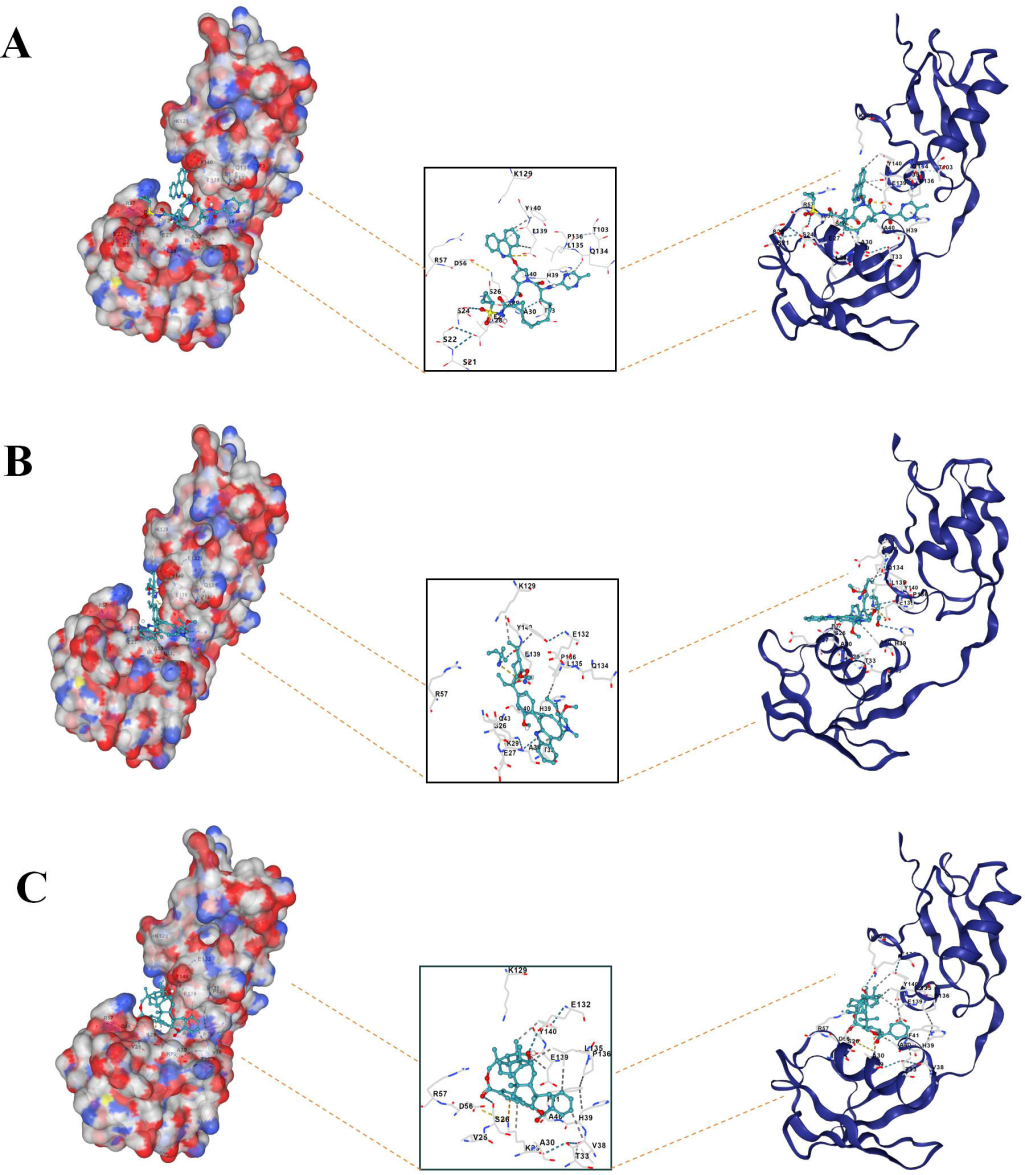
Molecular docking analysis of drug-gene interaction. **(A)** Molecular docking between ISG15 and paritaprevir. **(B)** Molecular docking between ISG15 and voacamine. **(C)** Molecular docking between ISG15 and 3, 29-dibenzoyl rarounitriol.

The screening process identified three compounds with significant docking scores, suggesting a strong binding affinity to ISG15: paritaprevir, an FDA-approved antiviral drug; voacamine, an investigational compound; and 3,29-dibenzoyl rarounitriol, derived from trichosanthes kirilowii maxim. Docking studies were conducted with the ISG15 protein to assess the binding potential of these compounds. **Figures 10A-C** display the 3D docking models, supporting the potential interaction between ISG15 and these molecules.

## Discussion

The clinical early-stage diagnosis of DM remains challenging due to its variable manifestations and the symptom overlap with other conditions. This is especially true for amyopathic DM, which often presents with a range of skin symptoms that can be easily confused with other inflammatory skin diseases and can lead to misdiagnosis or delayed diagnosis (18). Moreover, DM is associated with malignancies and severe complications such as pulmonary fibrosis that may cause death (2, 25). Hence, the identification of reliable biomarkers for DM is important for early diagnosis and treatment.

Our study applied a comprehensive suite of bioinformatics tools and machine learning algorithms to analyze gene expression datasets, resulting in the identification of ISG15 as a potential biomarker for DM. To our knowledge, this is the first study to confirm ISG15 as a robust diagnostic marker for DM in skin samples, exemplified by the AUC of 0.950 in ROC curve analysis.

Moreover, IHC findings confirmed the universally positive ISG15 expression in DM skin biopsies, particularly in perivascular regions and lymphocytes, areas known to be involved in DM pathology. Previous research by Salajegheh et al (12) indicated an overactive ISG15 conjugation pathway in DM muscle samples; however, muscle biopsies are more invasive and often encounter patient resistance due to the discomfort and potential complications. Detecting ISG15 in skin lesions may help physicians and patients to identify DM and distinguish it from other similar-looking inflammatory skin diseases. It could assist in the early diagnosis of DM, which is crucial for guiding their treatment and care strategies.

Several biomarkers, including anti-Jo-1 antibodies, anti-TIF-1γ antibodies, and anti-NXP-2 antibodies, are known for diagnosing DM (2). These markers are detected primarily through serological testing. However, the most frequently detected antisynthetase antibody in DM is anti-Jo-1 antibodies with a prevalence of 5–20% (26), indicating their limited diagnostic utility. Nevertheless, these biomarkers play a crucial role in understanding the disease’s pathophysiology. For instance, anti-Jo-1 antibodies are associated with associated with classic DM skin lesions, while anti- TIF-1γ (27) and anti-NXP-2^2^ antibodies are correlated with the risk of malignancy in DM. In comparison with these existing biomarkers, the strength of ISG15 lies in its high sensitivity and specificity in skin samples. This makes ISG15 a potentially useful marker, especially in clinically amyopathic DM or atypical DM patients, where muscle samples may not be readily available or exhibit atypical manifestations.

DM has traditionally been characterized as a humoral immune reaction-induced microangiopathy, mediated by complement and predominantly affecting the skin and muscles (28). The characteristic immunological profile in DM shows various immune cells around the blood vessels and within the connective tissue surrounding muscle fibers, primarily consisting of CD4 T cells, macrophages, and dendritic cells. While the cytotoxic CD8 T cells are the most prominent T cell subtype, a significant number of CD4 T cells are also present (28, 29). The KEGG enrichment of DEGs in pathways such as NOD-like receptor signaling and NF-kappa B signaling is consistent with previous findings that implicate innate immunity as a key player in DM pathophysiology (28). The immune effector processes and innate immune response highlighted by the GO analysis align with the known histopathological features of DM, including perivascular inflammation and the presence of lymphocytes and complement components in biopsies (30). Meanwhile, the significant immunostaining of ISG15 observed in the perivascular regions and lymphocytes within the skin tissue in our study indicated ISG15’s participation in those processes.

Our immune infiltration analysis revealed an enriched presence of specific immune cells, notably M1 macrophages and CD8 T cells, which likely contribute to the inflammatory and cytotoxic landscape of DM. The results also showed increased infiltration of resting dendritic cells, M0 macrophages, plasma cells, activated memory CD4 T cells and naive CD4 T cells in DM versus control skin samples. These cells likely contribute to DM pathogenesis via inflammatory and cytotoxic functions. The results consistent with the disease’s immune-mediated nature. To further understand the implications of these findings for the pathophysiology of DM, we delved deeper into the specific roles of these immune cell types in DM. For instance, M1 macrophages, known for their pro-inflammatory properties (31), may play a crucial role in tissue damage in DM, while the high infiltration of CD8 T cells underscores the importance of cytotoxic responses (32) in this condition. Additionally, the increase in resting dendritic cells and plasma cells may reflect the significant roles of antigen presentation and humoral immune responses in DM. The relationship between ISG15 expression and immune cells is also explored. It is found that ISG15 expression was positively correlated with M1 macrophages, monocytes, resting memory CD4 T cells, T follicular helper cells and dendritic cells. The data suggest a dual role for ISG15 in modulating the immune landscape in DM, in terms of M1 macrophages, when the infection or inflammation is severe enough to affect an organ, macrophages first exhibit the M1 phenotype to release TNF-α, IL-1β, IL-12, and IL-23 against the stimulus. But, if M1 phase continues, it can cause tissue damage (33). Meanwhile, the negative correlation between ISG15 and resting dendritic cells and plasma cells may indicate that ISG15 could inhibit antigen presentation and humoral immune responses. Based on the above analysis, ISG15 may have a dual role in DM: On one hand, ISG15 may exacerbate the inflammatory response in pathologic processes of DM progression by promoting the activity of M1 macrophages. Conversely, when the inflammatory response is overly pronounced, ISG15 may aid in averting an overactive or dysregulated immune response by modulating the functionality of specific immune cells. These findings provide new insights into the potential roles of ISG15 in DM, but further studies are needed to validate these hypotheses and elucidate the specific molecular mechanisms involved.

Furthermore, there’s a significant increase in activity within the type I IFN pathway, as observed in the muscle tissue and skin affected by DM^1^. Moreover, the severity of skin manifestations has been linked to the presence of a gene signature characteristic of type I IFN response (34). Among these genes, ISG15 stands out as a ubiquitin-like modifier that is strongly induced by type I IFN (13). Although the mechanisms leading to the induction of type I IFN in DM are not fully understood, increasing evidence points to the involvement of dendritic cells and subsequent Toll-like receptor (TLR) induction (10). This is consistent with our analysis results that ISG15 is significantly correlated with TLR signaling pathway and dendritic cells. ISG15’s role in DM appears to extend its known functions in immune modulation, as it is implicated in pathways governing protein turnover and cellular signaling (35). This is evidenced by its connection to ubiquitin-mediated proteolysis, as our GSEA analysis results showed, suggesting an active role for ISG15 in post-translational modifications, notably ISGylation (35). These pathways are vital for the maintenance of cellular stability and are believed to be tightly related to the pathogenic mechanisms of DM. Meanwhile, ISG15’s association with the regulation of cellular ion homeostasis and transport of cytosolic calcium ions reinforces its contribution to the complex network of cellular processes. Dysregulation of these processes could lead to an aggravated pathogenic state in DM, highlighting the potential of ISG15 as a biomarker and a therapeutic target in the disease.

Beyond diagnosis, ISG15’s association with various cancers, such as breast cancer and pancreatic cancer, provides insight into DM’s link with malignancies (36). Moreover, our findings suggest that ISG15 could play a crucial role in the pathogenesis of DM, providing a theoretical foundation for the development of diagnostic or therapeutic strategies based on ISG15. Firstly, measuring serum ISG15 levels could aid in the early diagnosis of DM and monitoring disease activity. Additionally, because ISG15 shows high expression in skin tissue samples, performing IHC detection of ISG15 during the early pathological examination of skin tissue can be highly indicative for early diagnosis, especially in clinically amyopathic DM or atypical DM patients, helping to avoid misdiagnosis and prevent disease progression. Modulating ISG15 expression or activity could represent a novel treatment avenue, especially for patients unresponsive to standard therapies. Our molecular docking analysis identified three compounds paritaprevir, voacamine, and 3,29-dibenzoyl rarounitriol as potential therapeutics, pending further validation. Nevertheless, as ISG15 is considered a “double-edged sword” for human diseases in which its expression is elevated, targeting ISG15 therapeutically requires caution due to its dual role in antiviral defense and immune regulation (35). Therapeutic strategies must balance diminishing the pathological immune response with maintaining innate antiviral defenses to minimize the risk of opportunistic infections. Future clinical studies should focus on validating these findings and establishing treatment protocols that ensure a balance between efficacy and safety in targeting ISG15.

However, there are some limitations to consider. First, the reliance on publicly available datasets introduces potential variability in sample collection, processing, and data quality. Second, confounding factors such as patient age, gender, and disease duration could have influenced our results. Third, relatively small sample size for the IHC validation is another limitation, larger cohorts would provide more definitive evidence of ISG15’s utility as a biomarker. Additionally, our study primarily focused on an Asian population, necessitating further validation across diverse populations. Future research should aim to address these limitations through larger, multi-center studies to validate ISG15 in skin biopsy samples as well as blood samples from various ethnic and geographic backgrounds. Moreover, functional studies, possibly involving animal models, are required to unravel the role of ISG15 in the inflammatory processes of DM.

## Conclusion

Our study not only validates ISG15 as a biomarker for DM but also sheds light on its potential role in the disease’s pathophysiology. Implementing IHC for ISG15 on skin biopsy specimens from lesions in individuals suspected of DM could prove to be a valuable diagnostic tool. The correlation of ISG15 with immune cell populations and its involvement in key biological processes and pathways provide a basis for further investigation into its utility in clinical practice.

## Data Availability

The datasets presented in this study can be found in online repositories. The names of the repository/repositories and accession number(s) can be found in the article/**Supplementary Material**.

## References

[1] Hornung T Wenzel J. Innate immune-response mechanisms in dermatomyositis: an update on pathogenesis, diagnosis and treatment. Drugs. (2014) 74:981–98. doi: 10.1007/s40265-014-0240-6 24939511

[2] Schlecht N Sunderkötter C Niehaus S Nashan D. Update on dermatomyositis in adults. JDDG: J der Deutschen Dermatologischen Gesellschaft. (2020) 18:995–1013. doi: 10.1111/ddg.14267 32985813

[3] Sasaki H Kohsaka H. Current diagnosis and treatment of polymyositis and dermatomyositis. Modern Rheumatol. (2018) 28:913–21. doi: 10.1080/14397595.2018.1467257 29669460

[4] Iaccarino L Ghirardello A Bettio S Zen M Gatto M Punzi L. The clinical features, diagnosis and classification of dermatomyositis. J Autoimmun. (2014) 48-49:122–7. doi: 10.1016/j.jaut.2013.11.005 24467910

[5] Miller FW Cooper RG Vencovský J Rider LG Danko K Wedderburn LR. Genome-wide association study of dermatomyositis reveals genetic overlap with other autoimmune disorders. Arthritis Rheumatism. (2013) 65:3239–47. doi: 10.1002/art.v65.12 PMC3934004 23983088

[6] Field MA. Detecting pathogenic variants in autoimmune diseases using high-throughput sequencing. Immunol Cell Biol. (2021) 99:146–56. doi: 10.1111/imcb.12372 PMC7891608 32623783

[7] Gao S Luo H Zhang H Zuo X Wang L Zhu H. Using multi-omics methods to understand dermatomyositis/polymyositis. Autoimmun Rev. (2017) 16:1044–8. doi: 10.1016/j.autrev.2017.07.021 28778709

[8] Wong D Kea B Pesich R Higgs BW Zhu W Brown P. Interferon and biologic signatures in dermatomyositis skin: specificity and heterogeneity across diseases. PloS One. (2012) 7:e29161. doi: 10.1371/journal.pone.0029161 22235269 PMC3250414

[9] Pinal-Fernandez I Casal-Dominguez M Derfoul A Pak K Plotz P Miller FW. Identification of distinctive interferon gene signatures in different types of myositis. Neurology. (2019) 93:e1193–204. doi: 10.1212/wnl.0000000000008128 PMC6808530 31434690

[10] Baechler EC Bilgic H Reed AM. Type I interferon pathway in adult and juvenile dermatomyositis. Arthritis Res Ther. (2011) 13:249. doi: 10.1186/ar3531 22192711 PMC3334651

[11] Preuße C Allenbach Y Hoffmann O Goebel HH Pehl D Radke J. Differential roles of hypoxia and innate immunity in juvenile and adult dermatomyositis. Acta Neuropathologica Commun. (2016) 4:45. doi: 10.1186/s40478-016-0308-5 PMC4847347 27121733

[12] Salajegheh M Kong SW Pinkus JL Walsh RJ Liao A Nazareno R. Interferon-stimulated gene 15 (*ISG15*) conjugates proteins in dermatomyositis muscle with perifascicular atrophy. Ann Neurol. (2010) 67:53–63. doi: 10.1002/ana.21805 20186858 PMC2875060

[13] Jeon YJ Yoo HM Chung CH. ISG15 and immune diseases. Biochim Biophys Acta (BBA) - Mol Basis Dis. (2010) 1802:485–96. doi: 10.1016/j.bbadis.2010.02.006 PMC7127291 20153823

[14] DeWane ME Waldman R Lu J. Dermatomyositis: Clinical features and pathogenesis. J Am Acad Dermatol. (2020) 82:267–81. doi: 10.1016/j.jaad.2019.06.1309 31279808

[15] Bohan A Peter JB. Polymyositis and dermatomyositis. New Engl J Med. (1975) 292:403–7. doi: 10.1056/NEJM197502202920807 1089199

[16] Bohan A Peter JB. Polymyositis and dermatomyositis. New Engl J Med. (1975) 292:344–7. doi: 10.1056/NEJM197502132920706 1090839

[17] Lundberg IE Tjärnlund A Bottai M Werth VP Pilkington C de Visser M. European league against rheumatism/american college of rheumatology classification criteria for adult and juvenile idiopathic inflammatory myopathies and their major subgroups. Arthritis Rheumatol. (2017) 2017:2271–82. doi: 10.1002/art.v69.12 PMC5846474 29106061

[18] Waldman R DeWane ME Lu J. Dermatomyositis: diagnosis and treatment. J Am Acad Dermatol. (2020) 82:283–96. doi: 10.1016/j.jaad.2019.05.105 31279813

[19] Taminau J Meganck S Lazar C Steenhoff D Coletta A Molter C. Unlocking the potential of publicly available microarray data using inSilicoDb and inSilicoMerging R/Bioconductor packages. BMC Bioinf. (2012) 13:335. doi: 10.1186/1471-2105-13-335 PMC3568420 23259851

[20] Cheung-Lee WL Link AJ. Genome mining for lasso peptides: past, present, and future. J Ind Microbiol Biotechnol. (2019) 46:1371–9. doi: 10.1007/s10295-019-02197-z PMC6989040 31165971

[21] Sanz H Valim C Vegas E Oller JM Reverter F. SVM-RFE: selection and visualization of the most relevant features through non-linear kernels. BMC Bioinf. (2018) 19:432. doi: 10.1186/s12859-018-2451-4 PMC6245920 30453885

[22] Xu W Liu X Leng F Li W. Blood-based multi-tissue gene expression inference with Bayesian ridge regression. Bioinformatics. (2020) 36:3788–94. doi: 10.1093/bioinformatics/btaa239 32277818

[23] Langfelder P Horvath S. WGCNA: an R package for weighted correlation network analysis. BMC Bioinf. (2008) 9:559. doi: 10.1186/1471-2105-9-559 PMC2631488 19114008

[24] Chen B Khodadoust MS Liu CL Newman AM Alizadeh AA. Profiling Tumor Infiltrating Immune Cells with CIBERSORT. Methods Mol Biol. (2018) 1711:243–59. doi: 10.1007/978-1-4939-7493-1_12 PMC5895181 29344893

[25] Mathai SC Danoff SK. Management of interstitial lung disease associated with connective tissue disease. BMJ. (2016) 352:h6819. doi: 10.1136/bmj.h6819 26912511 PMC6887350

[26] Satoh M Tanaka S Ceribelli A Calise SJ Chan EK. A comprehensive overview on myositis-specific antibodies: new and old biomarkers in idiopathic inflammatory myopathy. Clin Rev Allergy Immunol. (2017) 52:1–19. doi: 10.1007/s12016-015-8510-y PMC5828023 26424665

[27] Cobos GA Femia A Vleugels RA. Dermatomyositis: an update on diagnosis and treatment. Am J Clin Dermatol. (2020) 21:339–53. doi: 10.1007/s40257-020-00502-6 32096127

[28] Thompson C Piguet V Choy E. The pathogenesis of dermatomyositis. Br J Dermatol. (2018) 179:1256–62. doi: 10.1111/bjd.2018.179.issue-6 28542733

[29] Engel AG Arahata K. Mononuclear cells in myopathies: Quantitation of functionally distinct subsets, recognition of antigen-specific cell-mediated cytotoxicity in some diseases, and implications for the pathogenesis of the different inflammatory myopathies. Hum Pathol. (1986) 17:704–21. doi: 10.1016/s0046-8177(86)80180-0 3459704

[30] Emslie-Smith AM Engel AG. Microvascular changes in early and advanced dermatomyositis: A quantitative study. Ann Neurol. (1990) 27:343–56. doi: 10.1002/ana.410270402 2353792

[31] Quero L Tiaden AN Hanser E Roux J Laski A Hall J. miR-221-3p drives the shift of M2-macrophages to a pro-inflammatory function by suppressing JAK3/STAT3 activation. Front Immunol. (2020) 10:3087. doi: 10.3389/fimmu.2019.03087 32047494 PMC6996464

[32] Mittrücker H-W Visekruna A Huber M. Heterogeneity in the differentiation and function of CD8+ T cells. Archivum Immunologiae Therapiae Experimentalis. (2014) 62:449–58. doi: 10.1007/s00005-014-0293-y 24879097

[33] Shapouri-Moghaddam A Mohammadian S Vazini H Taghadosi M Esmaeili SA Mardani F. Macrophage plasticity, polarization, and function in health and disease. J Cell Physiol. (2018) 233:6425–40. doi: 10.1002/jcp.v233.9 PMC13482560 29319160

[34] Greenberg SA Higgs BW Morehouse C Walsh RJ Kong SW Brohawn P. Relationship between disease activity and type 1 interferon- and other cytokine-inducible gene expression in blood in dermatomyositis and polymyositis. Genes Immun. (2012) 13:207–13. doi: 10.1038/gene.2011.61 21881594

[35] Mirzalieva O Juncker M Schwartzenburg J Desai S. ISG15 and ISGylation in human diseases. Cells. (2022) 11:538. doi: 10.3390/cells11030538 35159348 PMC8834048

[36] Nguyen H-M Gaikwad S Oladejo M Agrawal MY Srivastava SK Wood LM. Interferon stimulated gene 15 (ISG15) in cancer: An update. Cancer Lett. (2023) 556:216080. doi: 10.1016/j.canlet.2023.216080 36736853

